# Supplementary material for: RBMP2 shapes specialized membranes for CO2 delivery in the pyrenoid condensate
Source: bioRxiv. 2026 Jul 3:2026.06.29.735335. Preprint. [Version 2] doi: 10.64898/2026.06.29.735335 (PMC13345187; doi:10.64898/2026.06.29.735335)
Supplement: Supplement 1 [file NIHPP2026.06.29.735335v2-supplement-1.pdf]

bioRxiv preprint doi: https://doi.org/10.64898/2026.06.29.735335; this version posted July 3, 2026. The copyright holder for this preprint (which was not certified by peer review) is the author/funder, who has granted bioRxiv a license to display the preprint in perpetuity. It is made available under a CC-BY 4.0 International license.

Chlamydomonas Resource Center ID	Strain Description	Mating type (mt)	Source	Antibiotic resistance
CC-5325	*CMJ030 (CC-5325)*	Minus (mt-)	Wildtype strain	none
CC-124	*CC-124*	mt-	Wildtype strain	none
LMJ.RY0402.215371	*rbmp2*	mt-	CLiP library LMJ.RY0402.215371	Paromomycin
CC-5647	*CMJ030; RBMP2-Venus-3xFLAG*	mt-	Transformation of CMJ030 with linearized RBMP2-Venus-3xFLAG. Previously published	Paromomycin
CC-6411	*rbmp2;RBMP2-Venus-3xFLAG*	mt-	Co-transformation of *rbmp2* mutant with linearized RBMP2-Venus-3xFLAG and hygromycin resistance gene	Paromomycin, hygromycin
CC-6412	*rbmp2;RBMP2-ΔRHO-Venus-3xFLAG*	mt-	Transformation of *rbmp2* mutant with linearized RBMP2-ΔRHO-Venus-3xFLAG	Paromomycin, hygromycin
CC-6413	*rbmp2;RBMP2-ΔMCP-Venus-3xFLAG*	mt-	Transformation of *rbmp2* mutant with linearized RBMP2-ΔMCP-Venus-3xFLAG	Paromomycin, hygromycin
CC-6414	*rbmp2;RBMP2-ΔTM-Venus-3xFLAG*	mt-	Transformation of *rbmp2* mutant with linearized RBMP2-ΔTM-Venus-3xFLAG	Paromomycin, hygromycin
CC-6415	*rbmp2;RBMP2-ΔRBM-Venus-3xFLAG*	mt-	Transformation of *rbmp2* mutant with linearized RBMP2-ΔRBM-Venus-3xFLAG	Paromomycin, hygromycin
LMJ.RY0402.174216	*cah3*	mt-	CLiP library LMJ.RY0402.174216	Paromomycin

Supplementary Table 1 | Chlamydomonas strains used in this study.

List of Chlamydomonas strains used in this study and their sources. CMJ030 is the wild-type background strain used for the CLiP mutant library. The *rbmp2* and *cah3* mutants were generated in CLiP mutant library study through insertional mutagenesis with a paromomycin-resistance cassette and were obtained from the CLiP library[46]. CC-5647 and CC-124 were described previously[27,47]. New strains generated in this study were produced by transformation.

bioRxiv preprint doi: https://doi.org/10.64898/2026.06.29.735335; this version posted July 3, 2026. The copyright holder for this preprint (which was not certified by peer review) is the author/funder, who has granted bioRxiv a license to display the preprint in perpetuity. It is made available under a CC-BY 4.0 International license.

Plasmid	Source	Chlamydomonas antibiotic resistance	*E. Coli* antibiotic resistance	Restriction enzymes
RBMP2-Venus-3xFLAG, pLM155	Previously published	Paromomycin	Kanamycin	XbaI
RBMP2-ΔRHO-Venus-3xFLAG, pHW013	Commercially synthesized RBMP2 lacking the rhodanese domain from pLM155 and switched with the Ori and antibiotic resistance genes from pRAM118	Hygromycin	Ampicillin	NdeI
RBMP2-ΔMCP-Venus-3xFLAG, pHW008	Commercially synthesized RBMP2 lacking the MCP domain from pLM155 and switched with the Ori and antibiotic resistance genes from pRAM118	Hygromycin	Ampicillin	NdeI
RBMP2-ΔTM-Venus-3xFLAG, pHW012	Commercially synthesized RBMP2 lacking the transmembrane domains from pLM155 and switched with the Ori and antibiotic resistance genes from pRAM118	Hygromycin	Ampicillin	NdeI
RBMP2-ΔRBM-Venus-3xFLAG, pHW010	Commercially synthesized RBMP2 lacking 6 Rubisco-binding motifs from pLM155 and switched with the Ori and antibiotic resistance genes from pRAM118	Hygromycin	Ampicillin	NdeI

Supplementary Table 2 | Plasmids used for Chlamydomonas transformation.

List of plasmids used for transformation into Chlamydomonas. The restriction enzyme used to linearize each plasmid before transformation is indicated. The pLM155 plasmid was generated and described in a previous study[27].

bioRxiv preprint doi: https://doi.org/10.64898/2026.06.29.735335; this version posted July 3, 2026. The copyright holder for this preprint (which was not certified by peer review) is the author/funder, who has granted bioRxiv a license to display the preprint in perpetuity. It is made available under a CC-BY 4.0 International license.

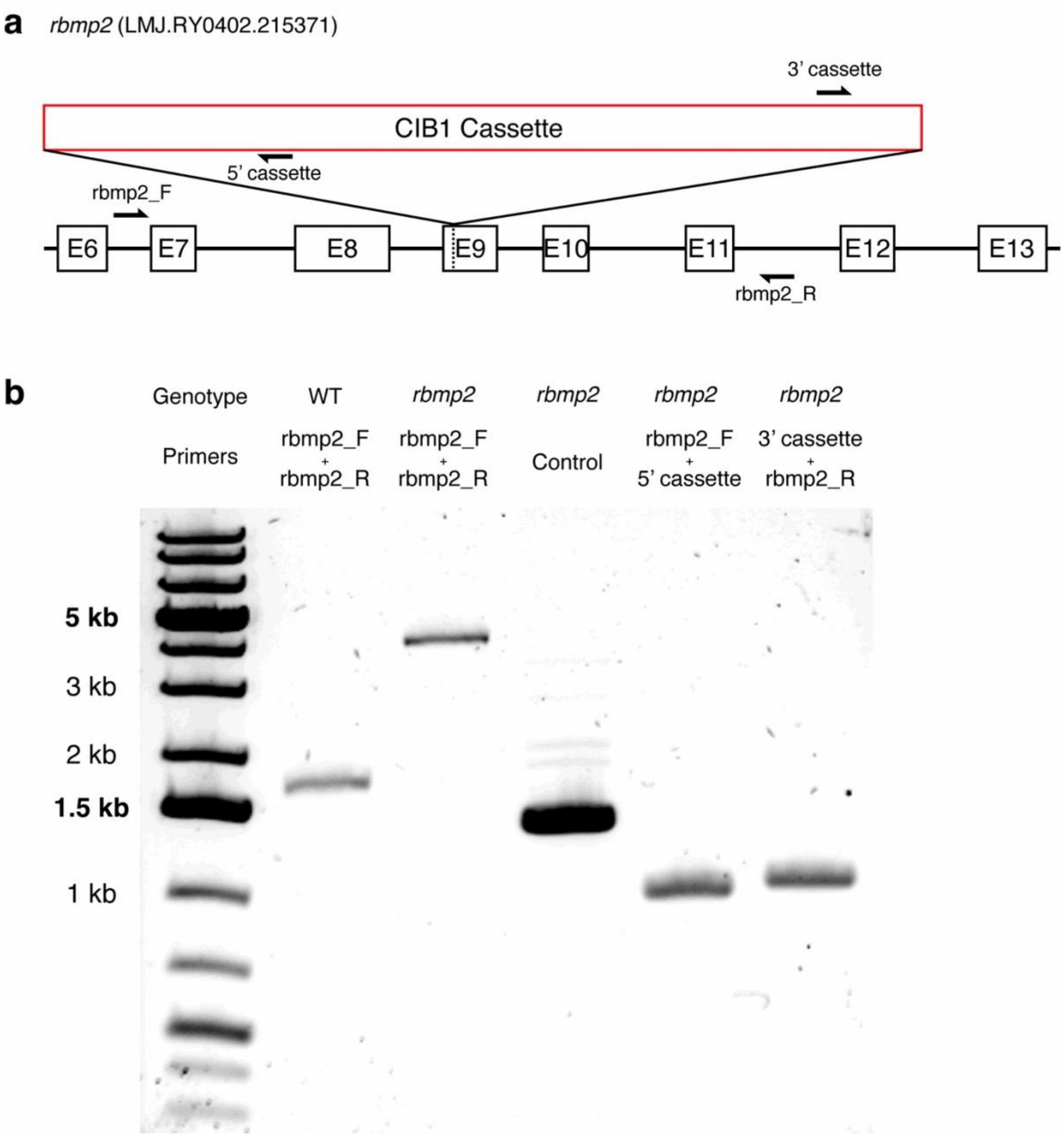

Supplementary Fig. 1 | PCR genotyping confirms the insertion in the rbmp2 mutant.

a, Schematic of the RBMP2 locus in the *rbmp2* mutant strain LMJ.RY0402.215371, showing the position of the CIB1 insertion cassette and the primers used for PCR genotyping. The CIB1 cassette is 2,223 bp. The exon primer pair *rbmp2_F/rbmp2_R* is expected to produce a 1,655-bp product from wild-type genomic DNA. The 5′ cassette primer pair *rbmp2_F/5′ cassette* is expected

bioRxiv preprint doi: https://doi.org/10.64898/2026.06.29.735335; this version posted July 3, 2026. The copyright holder for this preprint (which was not certified by peer review) is the author/funder, who has granted bioRxiv a license to display the preprint in perpetuity. It is made available under a CC-BY 4.0 International license.

to produce a 939-bp product from *rbmp2* genomic DNA, and the 3′ cassette primer pair *3′ cassette/rbmp2_R* is expected to produce a 973-bp product from *rbmp2* genomic DNA.

b, Agarose gel showing PCR amplification from genomic DNA isolated from wild-type and *rbmp2* cells. Amplification with rbmp2_F/rbmp2_R produced the expected wild-type product in wild type and a larger product in *rbmp2*, consistent with cassette insertion. PCRs using cassette-specific primers amplified the insertion junctions in *rbmp2*. Control primers were used to confirm genomic DNA quality.

bioRxiv preprint doi: https://doi.org/10.64898/2026.06.29.735335; this version posted July 3, 2026. The copyright holder for this preprint (which was not certified by peer review) is the author/funder, who has granted bioRxiv a license to display the preprint in perpetuity. It is made available under a CC-BY 4.0 International license.

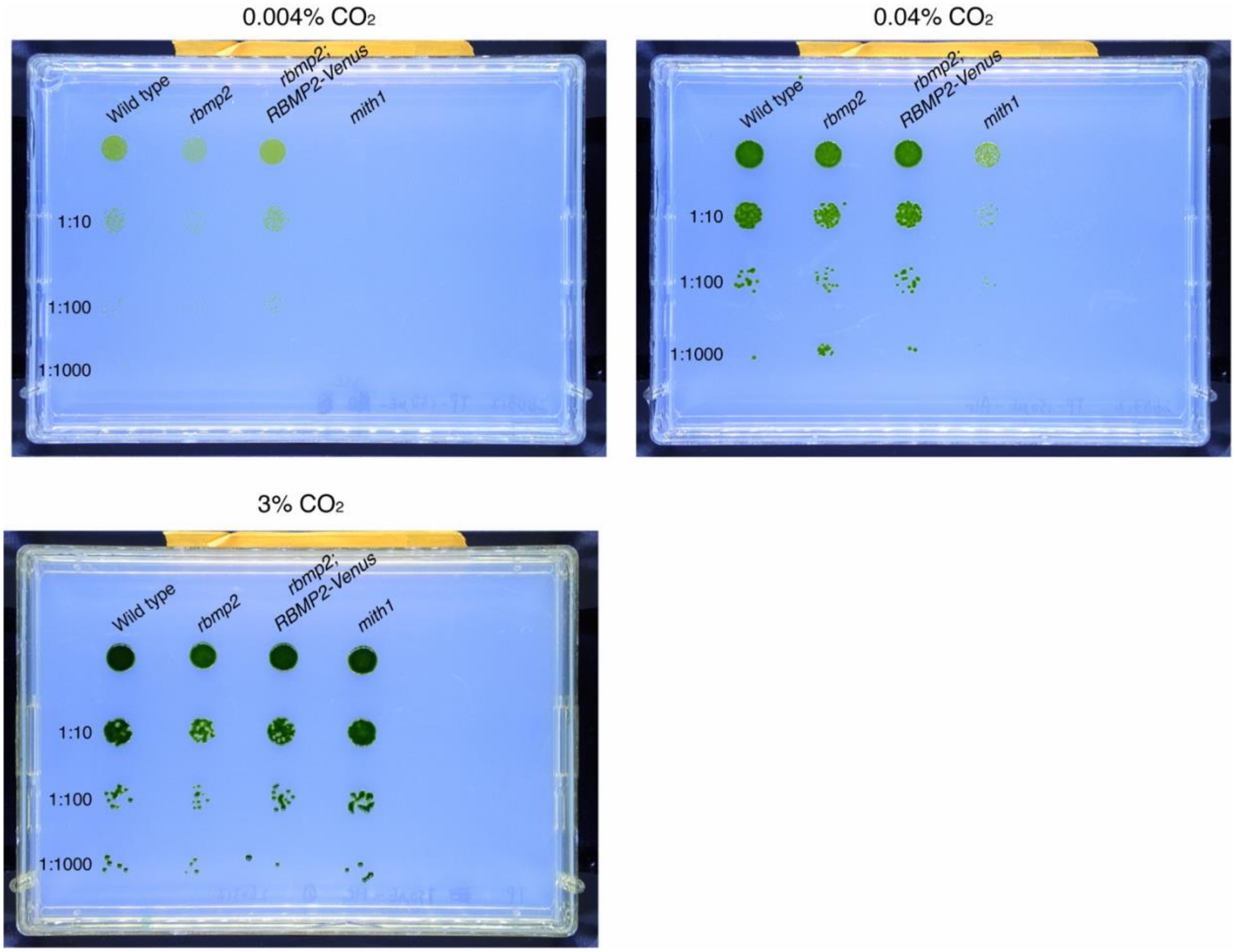

Supplementary Fig. 2 | Full plate images for the growth assay.

Full plate images corresponding to the spot growth assay shown in Fig. 1e. Cells were spotted on agar medium at the indicated serial dilutions and grown under very low CO_2, air-level CO_2, or high CO_2 conditions. Spot dilutions were 1:1, 1:10, 1:100 and 1:1000.

bioRxiv preprint doi: https://doi.org/10.64898/2026.06.29.735335; this version posted July 3, 2026. The copyright holder for this preprint (which was not certified by peer review) is the author/funder, who has granted bioRxiv a license to display the preprint in perpetuity. It is made available under a CC-BY 4.0 International license.

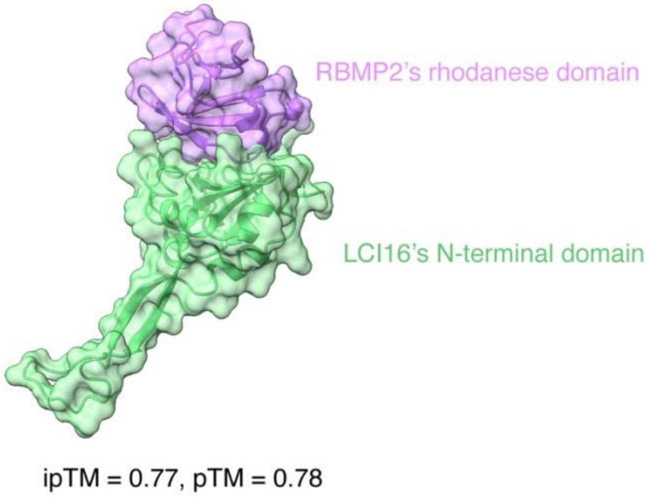

Supplementary Fig. 3 | AlphaFold2 predicts an interaction between LCI16 and the RBMP2 rhodanese domain.

AlphaFold2 prediction of the N-terminal domain of LCI16 bound to the rhodanese domain of RBMP2. The predicted complex is shown as a surface and cartoon representation, with LCI16 in green and the RBMP2 rhodanese domain in purple. The prediction had an ipTM score of 0.77 and a pTM score of 0.78.

bioRxiv preprint doi: https://doi.org/10.64898/2026.06.29.735335; this version posted July 3, 2026. The copyright holder for this preprint (which was not certified by peer review) is the author/funder, who has granted bioRxiv a license to display the preprint in perpetuity. It is made available under a CC-BY 4.0 International license.

Supplementary Video 1

Z-series of a wild-type (left) and an *rbmp2* mutant (right) pyrenoid combining anti-CAH3 immunofluorescence (green) with total protein stain (magenta) by ExM. Scale bars, ~1 µm, expansion corrected.

Supplementary Video 2

Tomograms of a wild-type (left) and an *rbmp2* mutant pyrenoid (right) by cryo-ET. Scale bar, 200 nm.

Supplementary Video 3

Z-series of an *rbmp2;ΔMCP-Venus* (left) and an *rbmp2;ΔTM-Venus* (right) pyrenoid combining anti-CAH3 immunofluorescence (green) with total protein stain (magenta) by ExM. Scale bars, ~1 µm, expansion corrected.

Supplementary Video 4

Tomograms of an *rbmp2;ΔMCP-Venus* (left) and an *rbmp2;ΔTM-Venus* (right) pyrenoid by cryo-ET. Scale bar, 200 nm.
